# Physiological and Pathological Mitochondrial Clearance Is Related to Pectoralis Major Muscle Pathogenesis in Broilers With Wooden Breast Syndrome

**DOI:** 10.3389/fphys.2020.00579

**Published:** 2020-06-16

**Authors:** Marina Hosotani, Takeshi Kawasaki, Yasuhiro Hasegawa, Yui Wakasa, Maki Hoshino, Naoki Takahashi, Hiromi Ueda, Tomohide Takaya, Tomohito Iwasaki, Takafumi Watanabe

**Affiliations:** ^1^Department of Veterinary Anatomy, School of Veterinary Medicine, Rakuno Gakuen University, Ebetsu, Japan; ^2^Research Office Concerning the Health of Humans and Birds, Abashiri, Japan; ^3^Department of Food Science and Human Wellness, College of Agriculture, Food and Environment Science, Rakuno Gakuen University, Ebetsu, Japan; ^4^Department of Agricultural and Life Science, Faculty of Agriculture, Shinshu University, Nagano, Japan

**Keywords:** wooden breast syndrome, pathology, hypoxia, mitochondria, autophagy, mitophagy

## Abstract

Wooden breast syndrome (WB) constitutes an emerging myopathy in the pectoralis major muscle (PM) of broiler chickens, characterized by myofiber hypertrophy and degeneration along with severe fibrosis. WB pathogenesis has been considered to involve hypoxia induced by rapid growth of the PM. In this study, we focused on mitochondrial morphology and dynamics in the myofibers, as these organelles are sensitive to damage by hypoxia, and examined the effects on WB pathogenesis. Specifically, the PMs of a flock of 35 broilers at 50 days of age were evaluated. First, the severity of disease in each bird was determined by measuring histopathological indices including the fibrotic area (FA) in the muscle and circularity of myofibers (CM). These values were 29.4 ± 9.6% and 0.70 ± 0.042, respectively, showing variety among the flock. Myofiber vacuolization was observed in all birds including numerous small- or large-rimmed vacuoles, with the former consisting of ultrastructurally autophagosome-like vacuoles engulfing degenerated mitochondria. The large-rimmed vacuoles frequently occurred in the PMs with more severe FA and CM, indicating a relationship between altered autophagy/mitophagy and WB severity. Next, the expression levels of hypoxia-adaptive and mitochondrial dynamics-related genes were analyzed, and their correlations with the histopathological indices were examined. The histopathological indices were negatively correlated with the expression of vascular endothelial growth factor A (*VEGFA*), indicating that less angiogenesis owing to weakened hypoxia-inducible factor signaling induces more severe WB pathology. In addition, the observed negative correlation with mitochondrial dynamics-related genes implied that WB pathology deteriorates concomitant with reduced mitochondrial dynamics. Furthermore, the expression of mitochondrial dynamics-related genes showed strong positive correlation with that of *VEGFA* and autophagy-/mitophagy-related genes. These results revealed that the PMs of broilers possess the mechanism of physiological clearance of mitochondria damaged by the hypoxia resulting from the continuous mitochondrial dynamics and autophagy/mitophagy accompanying rapid PM growth. In turn, the altered mitochondrial clearance induced by chronic hypoxia and the accumulation of damaged mitochondria likely underly the severe pathological features of WB.

## Introduction

In poultry production, meat quality impairment following the selective breeding of fast-growing broiler chickens necessary for supplying the large global demands of chicken meat represents an emerging problem worldwide (Kuttappan et al., 2013; Petracci et al., 2015). Specifically, genetic selection strategies have aimed to increase the yield of the pectoralis major muscle (PM), which constitutes the most superficial breast muscle (Bailey et al., 2015). However, the PMs of some broilers exhibit severe hardness, pale color, and small hemorrhages upon palpation and macroscopic observation in the poultry slaughterhouse (Sihvo et al., 2014) and ultimately cannot be utilized, which produces large economic losses (Abasht et al., 2016). This emerging myopathy, termed Wooden breast syndrome (WB) (Petracci et al., 2019), are characterized by histopathological features such as hypertrophy, necrosis and regeneration of myofibers, macrophage infiltration, and fibrosis (Sihvo et al., 2014; Clark and Velleman, 2016; Kawasaki et al., 2018). Consequently, the achievement of both high production efficiency of PM yield and reduction in the WB presentation is a highly desirable goal in the poultry industry.

In particular, broilers with the same genetics can show either no, mild, or severe WB symptoms at the same age even if raised under identical circumstances (Papah et al., 2018). WB severity is more highly associated with muscle development than with the overall growth of the body (Griffin et al., 2018). WB-affected PMs start to deteriorate after approximately 2 or 3 weeks (Radaelli et al., 2017; Kawasaki et al., 2018), such that a flock of broilers at later life would include birds at both initial and chronic stages of WB.

Wooden breast syndrome pathogenesis is considered to derive from insufficient angiogenesis during the rapid growth of the PMs leading to the production of oxidative stress and hypoxia (i.e., reduced oxygen availability) in the PMs, resulting in their myopathies (Mutryn et al., 2015; Malila et al., 2019). Hypoxia-inducible factor 1 (HIF1), which constitutes the primary transcriptional regulator produced in response to hypoxia in both physiological and pathological contexts, can increase vascularization and maintain oxygen homeostasis (Ziello et al., 2007; Thomas and Ashcroft, 2019). Specifically, HIF1-mediated cellular adaptations under hypoxic conditions regulate the metabolism, morphology, mass, and distribution of mitochondria through their fusion, fission, and autophagy/mitophagy (Thomas and Ashcroft, 2019). Notably, it has been reported that the expression of HIF1 transcripts and related genes is upregulated in the PMs of broilers with both moderate and severe WB compared to that of unaffected birds (Malila et al., 2019), with the first molecular signs of WB development being observed from 2 weeks of age (Papah et al., 2018). Nevertheless, different birds may exhibit individual susceptibilities to the development of such defects, as some are likely able to better counteract the development of hypoxic conditions giving rise to the cascade of events responsible for symptom development. As oxygen is essential for mitochondrial metabolism, we thus hypothesized that the damage to mitochondria mediated by hypoxia serves as the first step in WB pathogenesis, followed by muscle pathology such as hypertrophy and fibrosis. To evaluate the effect of mitochondrial damage on WB pathogenesis, in this study, we therefore performed PM histopathology and mitochondrial morphology assessments and analyzed the expression levels of mitochondrial dynamics-related genes in a flock of broilers including those with moderate to severe WB.

## Materials and Methods

### Animals and Specimens

Animal experimentation was approved by the Rakuno Gakuen University Institutional Animal Care and Use Committee (No. VH18A6) in accordance with the Act on Welfare and Management of Animals of the Japanese government. A total of 36 male chicks (Ross308) hatched in the university farm were raised until day 50. All birds were observed daily by animal stock technicians, and clinical conditions were checked by a poultry veterinarian as necessary during the rearing period. The birds were weighed and determined as clinically unaffected or exhibiting signs of WB by palpating the breast and via the simple method of wing lift examination, which can be performed in live birds by gently lifting up the wings of each bird to assess the ability to achieve back-to-back wing contact (Kawasaki et al., 2016) at 15, 44, and 50 days of age (just prior to euthanasia). The birds were housed in clean concrete-floored pen (1.8 × 1.2 m) covered with clean soft sawdust litter under suitable air conditioning and hygiene management and had free access to commercial corn-based diet and water. At 50 days of age, 35 birds weighing over 3,300 g were selected. The body weights of selected birds were 4,090 ± 478 g. All birds were euthanized by exsanguination under deep anesthesia induced using 20–30 mg/kg pentobarbital sodium into the radial vein with sterilized disposable 2.5-ml syringes and 23-gauge needles. The muscle samples were collected from one-third of the anterior part of the PM where fibrosis frequently occurs (Clark and Velleman, 2016; Hasegawa et al., 2020), fixed with Bouin’s fixative (Polysciences, Inc., Warrington, PA, United States) at 4°C for histological analysis, fixed with half-Karnovsky’s fixative [2.5% glutaraldehyde, 2% paraformaldehyde, 0.1 M cacodylate buffer (CB), pH 7.4] at 4°C for ultrastructural analysis, or immersed in RNAlater (Qiagen, Hilden, Germany) at 4°C for the analysis of gene expression. The PMs fixed with Bouin’s fixative were embedded in paraffin and cut into 4-μm-thick sections, which were then used for hematoxylin–eosin (HE) and Azan staining.

### Histoplanimetry of the PM

To evaluate the indices of muscle pathology, fibrotic area (FA) in the muscle and circularity of myofibers (CM) were measured. Several previous studies have reported that the primary histopathological characteristics in WB include collagen deposition (fibrosis) in the interstitium and lack of polygonality of myofibers (Sihvo et al., 2014; Kawasaki et al., 2018). Therefore, we chose the FA and CM as representative histopathological parameters to show the degree of WB-mediated deterioration. To calculate the FA, the area of myofibers (*a*) and the whole area of muscle (*b*) in four different randomly selected regions of individual Azan-stained histological sections of each specimen were measured using Image Pro software V10.0.04 (Media Cybernetics, Inc., Rockville, MD, United States). The FA was calculated as follows: FA (%) = 100 × [whole area of the muscle (*b*) - area of myofibers (*a*)]/whole area of the muscle (*b*). According to the FA value, the birds were divided into stages I–VI by groups of 5% units; i.e., stage I (<20%, *n* = 6), stage II (20–25%, *n* = 5), stage III (25–30%, *n* = 6), stage IV (30–35%, *n* = 5), stage V (35–40%, *n* = 9), and stage VI (>40%, *n* = 4). To calculate the CM, 200 myofibers on four different randomly selected regions of individual HE-stained histological sections of each specimen were manually traced, and their CMs were calculated using ImageJ ver1.52a (National Institutes of Health, Bethesda, MD, United States). The formula for CM calculation is 4*p* × area of myofiber/(perimeter of myofiber)^2^.

### Ultrastructural Analysis

For transmission electron microscope (TEM) analysis, the PMs of sample numbers 1, 2, 6, 9, 10, 15, 17, 19, and 33 (*n* = 9) fixed by half-Karnovsky’s fixative were used for specimen preparation. The contrast of the mitochondrial membrane structures was enhanced for TEM by heavy metal block staining, as described previously (Thai et al., 2016). Briefly, the fixed PMs were washed four times with a solution containing 0.1 M CB (pH 7.4) and 2% OsO_4_ (TAAB Laboratories Equipment Ltd., Berks, United Kingdom) in 0.15% K_4_(CN)_6_ (Nacalai Tesque, Inc., Kyoto, Japan) for 4 min each at 4°C and soaked in the same solution for 1 h. After four washes with distilled water (4 min each), the samples were immersed in 0.1% thiocarbohydrazide (Sigma Aldrich Co. LLC., St. Louis, MO, United States) for 20 min at room temperature, then washed again with distilled water and immersed in 2% OsO_4_ for 30 min at room temperature. Following further distilled water washing, the samples were immersed in 1% uranyl acetate at 4°C overnight, washed again in distilled water, and immersed in Walton’s lead aspartate solution at 60°C for 30 min. Finally, the samples were dehydrated through an ethanol series, transferred to QY-1, and embedded in epoxy resin (Quetol 812; Nissin EM Co. Ltd., Tokyo, Japan). The tissue block was sliced into 80-nm-thick sections perpendicular to the long axis of the myofiber using an ultramicrotome (JUM-7; JEOL Ltd., Tokyo, Japan). The sections were collected on a 100-mesh copper grid, and the structures were observed by TEM (HT-7700; Hitachi High Technology Co., Tokyo, Japan) at an acceleration voltage of 80 kV.

### Reverse Transcription and Quantitative Real-Time Polymerase Chain Reaction

The PMs of broilers were homogenized using a BioMasher (Nippi, Inc., Tokyo, Japan), and total RNA from samples was purified using TRIzol reagent (Life Technologies, Carlsbad, CA, United States) according to the manufacturer’s instructions. The purified total RNA was used as a template to synthesize complementary DNA (cDNA) using ReverTra Ace qPCR RT Master Mix (Toyobo Co., Ltd., Osaka, Japan). Quantitative real-time PCR (qPCR) analysis was performed on the cDNA using THUNDERBIRD SYBR qPCR Mix (Toyobo Co., Ltd.) and gene-specific primers (Table 1, Sigma-Aldrich). The qPCR cycling conditions were 95°C for 1 min, followed by 40 cycles of 95°C for 15 s, and 60°C for 45 s. Data were normalized against the expression level of actin, beta (*ACTB*) and analyzed using the ΔCt method for correlation analysis and the ΔΔCt method for stage-related comparison of gene expression.

**TABLE 1:** List of primers used in the study.

**Gene**	**Accession number**	**Primer sequence (5′–3′) F: Forward, R: Reverse**	**Product size (bp)**
*ACTB*	NM_205518.1	F: ACAATGGCTCCGGTATGTG	133
		R: CCAACGTAGCTGTCTTTCTGG	
*DRP1*	NM_001079722.1	F: GGTGATCAACAAGCTGCAAGA	180
		R: GCACCAGCTGCAGAATAAGG	
*HIF1A*	NM_204297.1	F: GTCACCGACAAGAAGAGGATTAG	225
		R: TTCTGTCTCTAGCTCACCAGCA	
*MAP1LC3A*	XM_417327.6	F: GTACAGCAGATCCGAGAGCA	298
		R: AGGTCTCCTGGGAAGCGTAG	
*MAP1LC3B*	NM_001031461.1	F: ACGTCAACATGAGCGAGCTA	197
		R: TTGCACTCCGAAAGTCTCCT	
*MFN1*	NM_001012931.2	F: TGGGATTGGCCATACAACTAAC	277
		R: GAATTTGTCAATCCAGCTGTCC	
*MFN2*	XM_015297205.2	F: TGATGGGCTATAACGACCAGA	208
		R: GAGAGAGCAATCAGCCTCCA	
*OPA1*	NM_001039309.1	F: TGATTGCTCAAGCCCGAATA	250
		R: CCAGGACCTCTCACACTTAAGGA	
*PARK2*	XM_419615.6	F: GGAGAAGAACAGTACAACCGCTAC	182
		R: CATTCACGGCAGAACACAA	
*VEGFA*	NM_205042.2	F: ACAATTGAGACCCTGGTGGA	195
		R: CGCTATGTGCTGACTCTGATG	

**ACTB*, actin, beta; *DRP1*, dynamin-related protein 1; *HIF1A*, hypoxia inducible factor 1 subunit alpha; *MAP1LC3A, B*, microtubule associated protein 1 light chain 3 alpha, beta; *MFN1, 2*, mitofusion 1, 2; *OPA1*, OPA1, mitochondrial dynamin like GTPase; *PARK2*, parkin RBR E3 ubiquitin protein ligase; *VEGFA*, vascular endothelial growth factor A.*

### Statistical Analysis

Results were expressed as the mean ± standard error (SE). Data among three or more groups were compared using the Tukey test (*P* < 0.05). Data between two groups were compared using the Student’s *t* test (*P* < 0.05). Correlations between two parameters were analyzed using Spearman’s correlation test (*P* < 0.05).

## Results

### Histopathological Observation of the PM

**FIGURE 1:**
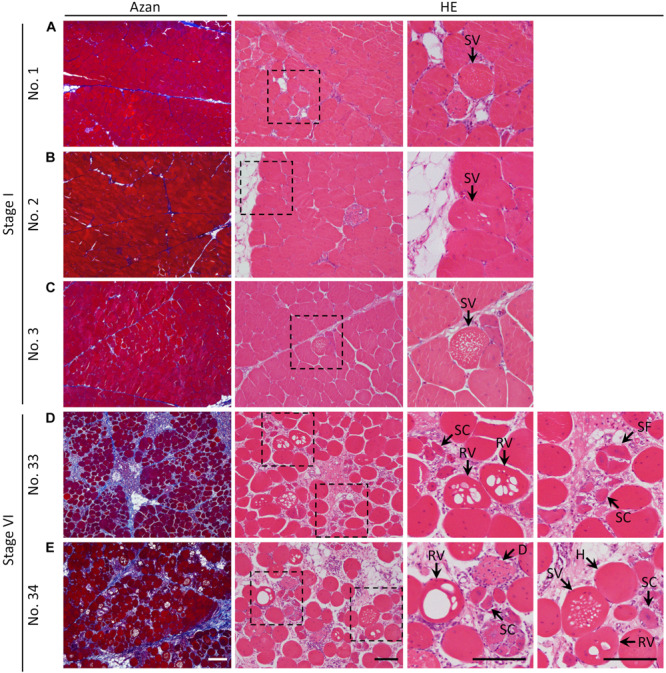
Histology of the pectoralis major muscle of broilers stained with Azan and hematoxylin–eosin. **(A–E)** Histological observations of the pectoralis major muscle of **(A)** sample number 1 (stage I; FA, 7.24% and CM, 0.70), **(B)** 2 (stage I; FA, 9.06% and CM, 0.69), **(C)** 3 (stage I; FA, 13.25% and CM, 0.68), **(D)** 33 (stage VI; FA, 40.90% and CM, 0.70), and **(E)** 34 (stage VI; FA, 40.97% and CM, 0.76). The squares indicated by dashed lines are magnified on the right. White bar = 200 mm, black bar = 100 mm. SV, myofiber with numerous small vacuoles; RV, myofiber with large-rimmed vacuoles; H, hypertrophy of myofiber; SC, myofiber with small caliber; SF, split fiber; D, myofiber degeneration; FA, fibrotic area in the muscle; CM, circularity of myofibers.

As shown in Figure 1, the histological sections of the PMs stained with Azan and HE showed both mild and severe fibrotic pathology, as revealed by the collagen distribution stained blue with Azan, and myofiber hypertrophy. The values of FA and CM in the PMs of each broiler are shown in Table 2; mean values in all birds were 29.4 ± 9.6% and 0.70 ± 0.042%, respectively. According to FA, the birds were grouped into six stages. The CM tended to increase with higher stage (Table 2) and significantly positively correlated with the FA [Spearman’s correlation coefficient (*ρ*) = 0.6246, *P* < 0.0001]. In PMs with relatively low levels of FA and CM among all broilers (stage I), not only the normal polygonal myofibers but also myofibers with numerous small vacuoles were observed (Figures 1A–C). In comparison, in PMs with relatively high levels of FA and CM (stage VI), several types of myofibers with pathology could be seen: myofibers with numerous small vacuoles and large-rimmed vacuoles, degenerated myofibers, myofibers with small caliber, and split fibers (Figures 1D,E). During the observation period, we obtained clinical information including body weight at 15, 44, and 50 days, weight gain ratio from 15 to 50 days, and results of palpation and wing lift examination (Supplementary Table 1). Comparison and correlation analysis between PM histopathology and these clinical data were performed; however, no significant associations were detected (Supplementary Tables 2, 3).

**TABLE 2:** The fibrotic area (FA) in muscle, circularity of myofibers (CM), and stage classified by the FA in each bird.

**Sample No.**	**FA (%)**	**CM**	**Stage**	**Mean ± SE (%) of FA in each stage**	**Mean ± SE of CM in each stage**
1	7.24	0.70	I	13.45 ± 1.81^II, III, IV, V, VI^	0.67 ± 0.02^V^
2	9.06	0.69	I	–	–
3	13.25	0.68	I	–	–
4	16.31	0.67	I	–	–
5	17.10	0.68	I	–	–
6	17.76	0.57	I	–	–
7	21.53	0.67	II	23.59 ± 0.58^IV, V, VI^	0.68 ± 0.01
8	23.28	0.70	II	–	–
9	24.06	0.67	II	–	–
10	24.13	0.67	II	–	–
11	24.92	0.68	II	–	–
12	25.86	0.61	III	27.05 ± 0.64^IV, V, VI^	0.68 ± 0.02
13	26.15	0.74	III	–	–
14	26.19	0.66	III	–	–
15	26.28	0.74	III	–	–
16	27.95	0.70	III	–	–
17	29.86	0.66	III	–	–
18	31.14	0.73	IV	31.96 ± 0.51^V, VI^	0.70 ± 0.01
19	31.18	0.69	IV	–	–
20	31.35	0.68	IV	–	–
21	32.05	0.70	IV	–	–
22	34.08	0.72	IV	–	–
23	36.92	0.74	V	38.15 ± 0.35	0.74 ± 0.01
24	37.00	0.74	V	–	–
25	37.37	0.74	V	–	–
26	37.80	0.75	V	–	–
27	37.93	0.69	V	–	–
28	38.05	0.75	V	–	–
29	39.21	0.75	V	–	–
30	39.47	0.74	V	–	–
31	39.61	0.71	V	–	–
32	40.65	0.70	VI	40.88 ± 0.08	0.72 ± 0.01
33	40.90	0.70	VI	–	–
34	40.97	0.76	VI	–	–
35	41.00	0.72	VI	–	–

*Significant differences among stages are indicated by superscripted stage no. (I, II, III, IV, V, and VI) (*P* < 0.05, Tukey test).*

**TABLE 3:** Spearman’s correlation coefficient (*ρ*) between the hypoxia-adaptive gene expression and muscle histopathological indices.

**Gene**		**Histopathological index**	
		**FA**	**CM**
*HIF1A* (*n* = 35)	*ρ*	0.2616	0.2392
	*P*	0.129	0.1664
*VEGFA* (*n* = 35)	*ρ*	–0.4476	–0.584
	*P*	0.007**	0.0002***

*HIF1A*, hypoxia inducible factor 1 subunit alpha; *VEGFA*, vascular endothelial growth factor A; FA, fibrotic area in the muscle; CM, circularity of myofibers. **P < 0.01, ***P < 0.001.

### Ultrastructural Observation of PM Myofibers

**FIGURE 2:**
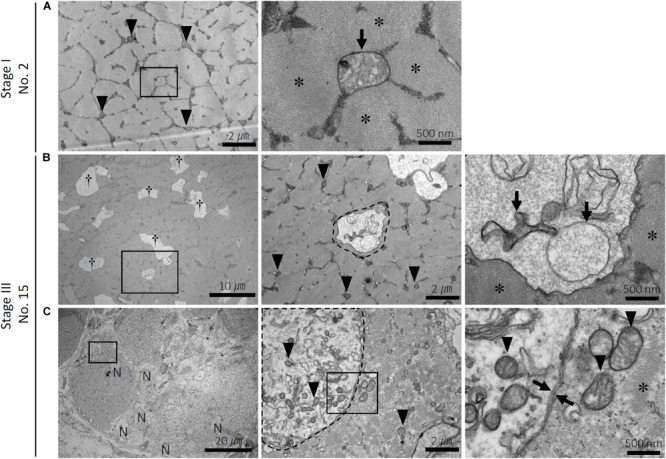
Ultrastructure of the myofibers in the pectoralis major muscle of broilers. Ultrastructure of the pectoralis major muscle of **(A)** sample number 2 (stage I; FA, 9.06% and CM, 0.69) and **(B,C)** 15 (stage III; FA, 26.28% and CM, 0.74). FA, fibrotic area in the muscle; CM, circularity of myofibers. **(A)** Distribution of small and clear mitochondria (arrowheads) and a swelled mitochondrion (arrow) within myofibrils (asterisks) in the normal myofiber. The square indicated by black line is magnified on the right. **(B)** Numerous small vacuoles (daggers) are distributed within myofibrils (asterisks) in the myofibers. The vacuole (surrounded by a dashed line) envelops the swelled and degenerated mitochondria (arrows). The square indicated by black line is magnified on the right. Arrowheads: small and clear mitochondria. **(C)** Multiple myoblasts accumulated in the pectoralis major muscle. The autophagosome with a double membrane (between arrows) in the myoblast envelops organelles and the small and clear mitochondria (arrowheads). The squares indicated by black line are magnified on the right. N, nuclei in the myoblast; asterisks, myofibrils.

In healthy myofibers, both normal, small mitochondria containing clear intrastructures and swelled round mitochondria were localized within the intact myofibrils, which are composed of myosin (Figure 2A). In the myofibers with numerous small vacuoles, spherical structures with a single-membrane containing degraded mitochondria, reflecting autophagosome-like vacuoles, were observed (Figure 2B). The myofibrils around the vacuole clearly showed intact morphology. Myofiber regeneration by fusion of multiple myoblasts was also observed (Figure 2C). In the cytosol of the myoblasts, spherical structures with a double-membrane containing normal mitochondria and other organelles such as endoplasmic reticulum, reflecting typical autophagosomes, were observed (Figure 2C).

### Relationship Between Hypoxia and Histopathological Observations in the PM

To examine the association of the muscle hypoxic conditions in WB with the histopathological observations such as fibrosis and hypertrophy in the myofibers, the correlations between the hypoxia-adaptive genes *HIF1A* and vascular endothelial growth factor A (*VEGFA*), and the muscle histopathological indices FA and CM were analyzed (Table 3). Although the expression of *HIF1A* did not show significant correlations with either histopathological index, that of *VEGFA* was significantly negatively correlated with both pathological indices.

### Relationship Between Mitochondrial Dynamics and Pathological Observations, Hypoxia, or Autophagy/Mitophagy in the PM

To reveal the association of the mitochondrial dynamics with the histopathological and hypoxic conditions in the PMs, the correlations between the expression of mitochondrial fusion- [mitofusion 1, 2 (*MFN1*, *MFN2*) and mitochondrial dynamin-like GTPase (*OPA1*)] and fission [dynamin-related protein 1 (*DRP1*)]-related genes and the histopathological indices (FA and CM) or the expression of hypoxia-adaptive genes *HIF1A* and *VEGFA* were analyzed (Table 4). The expression of *MFN1* and *MFN2* was significantly negatively correlated with CM and both FA and CM, respectively. Alternatively, *DRP1* and *HIF1A* expression significantly positively correlated. In addition, the expression of all mitochondrial dynamics-related genes including *MFN1*, *MFN2*, *OPA1*, and *DRP1* showed strong significantly positive correlation with that of *VEGFA*.

**TABLE 4:** Spearman’s correlation coefficient (*ρ*) between mitochondrial dynamics-related gene expression and muscle histopathological indices or hypoxia-adaptive and autophagy-/mitophagy-related gene expression.

**Gene**		**Parameter**						
		**Histopathological index**		**Hypoxia-adaptive gene expression**		**Autophagy-/mitophagy-related gene expression**		
		**FA**	**CM**	***HIF1A***	***VEGFA***	***MAP1LC3A***	***MAP1LC3B***	***PARK2***
*MFN1*	*ρ*	–0.279	–0.3364	0.2501	0.6804	0.5882	0.5832	0.565
(*n* = 35)	*P*	0.1046	0.0482*	0.1473	< 0.0001***	0.0002***	0.0002***	0.0004***
*MFN2*	*ρ*	–0.3734	–0.4451	0.0482	0.7555	0.6384	0.6563	0.4866
(*n* = 35)	*P*	0.0271*	0.0074**	0.7834	< 0.0001***	< 0.0001***	< 0.0001***	0.003**
*OPA1*	*ρ*	–0.1499	–0.2574	0.2714	0.5644	0.6339	0.5933	0.3854
(*n* = 35)	*P*	0.3902	0.1355	0.1147	0.0004***	< 0.0001***	0.0002***	0.0222*
*DRP1*	*ρ*	–0.2734	–0.237	0.3353	0.6204	0.688	0.6796	0.5232
(*n* = 35)	*P*	0.112	0.1705	0.049*	< 0.0001***	< 0.0001***	< 0.0001***	0.0013**

FA, fibrotic area in the muscle; CM, circularity of myofibers; *MFN1, 2*, mitofusion 1, 2; *OPA1*, OPA1, mitochondrial dynamin-like GTPase; *DRP1*, dynamin-related protein 1; *HIF1A*, hypoxia inducible factor 1 subunit alpha; *VEGFA*, vascular endothelial growth factor A; *MAP1LC3A, B*, microtubule associated protein 1 light chain 3 alpha, beta; *PARK2*, parkin RBR E3 ubiquitin protein ligase. * *P* < 0.05, ** *P* < 0.01, *** *P* < 0.001.

Based on the ultrastructural observations of the active metabolism of mitochondria as revealed by autophagy-like vacuoles, the correlations between the expression of mitochondrial dynamics-related genes and that of autophagy-/mitophagy-related genes were also analyzed (Table 4). Specifically, the microtubule-associated protein 1 light chain 3 alpha, beta (*MAP1LC3A*, *B*) genes, also termed LC3A and LC3B, constitute the primary genes related to autophagosome development and maturation (Schaaf et al., 2016), whereas parkin RBR E3 ubiquitin protein ligase (*PARK2*) acts as a specific regulator of mitophagy (Sato et al., 2018). We found that the expression of *MFN1*, *MFN2*, *OPA1*, and *DRP1* was significantly positively correlated with that of all three autophagy-/mitophagy-related genes.

**FIGURE 3:**
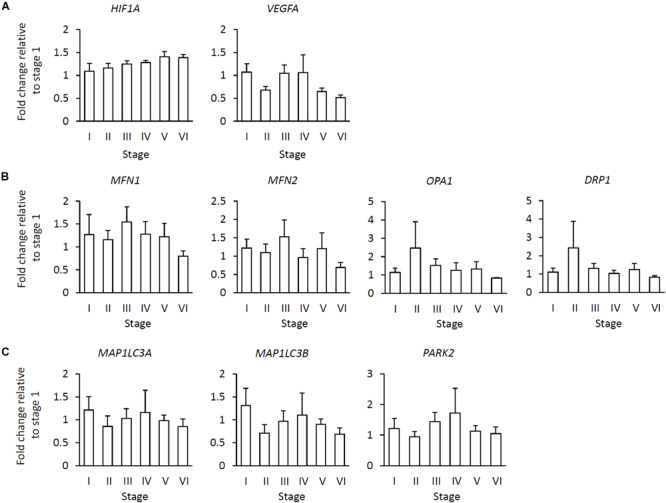
Relationship of gene expression levels in the various stages to fibrotic area in the muscle. **(A)** Hypoxia-adaptive gene expression including hypoxia inducible factor 1 subunit alpha (*HIF1A*) and vascular endothelial growth factor A (*VEGFA*). **(B)** Mitochondrial dynamics-related gene expression including mitofusion 1, 2 (*MFN1*, *2*), OPA1, mitochondrial dynamin-like GTPase (*OPA1*), and dynamin-related protein 1 (*DRP1*). **(C)** Autophagy-/mitophagy-related gene expression including microtubule-associated protein 1 light chain 3 alpha, beta (*MAP1LC3A*, *B*) and parkin RBR E3 ubiquitin protein ligase (*PARK2*). Data represent the means ± SE [stage I (*n* = 6), stage II (*n* = 5), stage III (*n* = 6), stage IV (*n* = 5), stage V (*n* = 9), and stage VI (*n* = 4), Tukey test (*P* < 0.05)].

We further compared the expression levels of all examined genes among the six stages. No significant stage-related differences were observed in the expression of any of the examined genes (Figures 3A–C).

## Discussion

The histology of broiler PMs revealed myofiber hypertrophy and internal fibrosis, consistent with previous reports (Sihvo et al., 2014; Velleman and Clark, 2015), whereas the values of FA and CM histopathological indices differed among individuals, revealing that WB severity in the broilers varies during later life. Myofiber vacuolization was observed in all PMs analyzed in this study. Myofibers with numerous small vacuoles were apparent in the PMs with both mild and more severe FA and CM, whereas those with large-rimmed vacuoles frequently occurred in the PMs with more severe index scores. These vacuoles are thus termed autophagic vacuoles (AVs) in skeletal muscle (Malicdan and Nishino, 2012), and their appearance therein constitutes the pathognomonic morphological hallmark of autophagic vacuolar myopathy, which is a group of human hereditary myopathies including Pompe and Danon disease (Nishino, 2006; Cho and Noguchi, 2013; Castets et al., 2016). It has been reported that rimmed vacuoles are outcomes of impaired normal autophagy and represent frequent pathological characteristics in the chronic muscle damage of atrophy and myopathies (Suzuki et al., 2002; Sato et al., 2018). During autophagy, the accumulation of protein aggregates disturbs the maturation and fusion steps of autophagosome formation and enlarges the autophagic vesicles, where the rimmed vacuoles originate (Castets et al., 2016). Therefore, the rimmed vacuoles frequently observed in the PMs of broilers with more severe FA and CM implies the pathological condition of autophagy, indicating the close relationship between the altered autophagy and WB severity.

The ultrastructural observations of mitochondrial morphology confirmed that both physiological and pathological autophagies occur in the broiler skeletal muscles. Even in the histologically healthy myofibers, not only normal but also swelled and degenerated mitochondria are distributed within the myofibrils. In the myofibers exhibiting numerous small vacuoles, the AVs appeared to engulf the degenerated mitochondria. Moreover, because these AVs were enveloped by a single membrane, it was inferred that they were in the degradation stage during autophagy, in which the autophagosome double membrane is degraded by lysosomes as an autolysosome (Eskelinen et al., 2011). In mouse studies, deficiency of apolipoprotein B messenger RNA (mRNA) editing enzyme catalytic polypeptide 2 (APOBEC2), a member of the zinc-dependent cytidine deaminase protein family, has been reported to lead to a loss of skeletal muscle mass and atrophy (Sato et al., 2009). Notably, APOBEC2 deficiency does not affect the sarcomeric structure but rather causes mitochondrial morphological abnormalities in the murine skeletal myofibers, with the abnormal mitochondria being surrounded by AVs and removed by mitophagy (Sato et al., 2018). In comparison, in the myofibers of broilers analyzed in the present study, the myofibrils surrounding the AVs were ultrastructurally healthy and intact, indicating that the engulfment and digestion of these vacuoles is limited to the mitochondria likely through the mitophagy process. Specifically, to maintain skeletal muscle homeostasis, muscle repair and remodeling promotes the proliferation of satellite cells as myoblasts, which activates their myogenic differentiation (Sin et al., 2016). The autophagy and/or mitophagy in myoblasts are physiologically required in order to regenerate the skeletal muscle by myoblast differentiation (Sin et al., 2016; Tokutake et al., 2019). Herein, we observed typical autophagosomes with double membranes engulfing organelles in the myoblasts of the broiler PM. Therefore, we consider that, in the broilers, the autophagy/mitophagy that occurs via AVs in the myofibers and via autophagosomes in the myoblasts has different biological interpretations.

Hypoxia-inducible factor1 is a heterodimer composed of an alpha and a beta subunit, which are respectively induced by hypoxia and constitutively expressed (Greijer and van der Wall, 2004). HIF1α mediates the primary transcriptional response to hypoxia by binding to conserved sequences in the promoter regions of various hypoxia-adaptive genes (Majmundar et al., 2010; Thomas and Ashcroft, 2019). VEGF is the principal transcriptional target of HIF1α and induces angiogenesis in the hypoxic condition, which increases the oxygen supply (Majmundar et al., 2010). In skeletal muscles, the hypoxia consequent to exercise and ischemia is known to induce angiogenesis through HIF1–VEGF signaling (Ohno et al., 2012). In the PMs of the broilers, *VEGFA* expression was significantly negatively correlated with the muscle pathological indices FA and CM, indicating that the decreased angiogenesis and oxygen supply in the skeletal muscle worsened the symptoms of WB. As hypoxia-mediated HIF1 induction occurs at the level of protein stability whereas *HIF1A* mRNA expression remains unchanged (Greijer and van der Wall, 2004), our correlation analysis results suggest that *HIF1A* expression and WB severity are not related in the broiler PMs.

Rather, the small caliber myofibers and split fibers observed in the PMs with more severe FA and CM, in which the angiogenesis is insufficient for supplying oxygen, are considered to be the outcomes of impaired myofiber regeneration consequent to the severe hypoxia. In general, the hypoxia-mediated impairment of skeletal muscle regeneration promotes the loss of muscle mass and atrophy (Neel et al., 2013; Romanello and Sandri, 2016). However, in broilers, the skeletal muscles develop fibrosis within the degenerated myofibers instead of decreasing their mass, resulting in the retention of high PM mass. In comparison, in the field of cancer medicine, it is recognized that following repeated and chronic periods of hypoxia, selection for resistance to the hypoxia-induced HIF1 response may occur, which exacerbates hypoxia-adaptive pathologies (Greijer and van der Wall, 2004). We consider that the chronic hypoxia in the broiler PMs might similarly alter the reactivity to HIF signaling and attenuate PM adaptability to the hypoxia.

Mitochondria undergo continuous cycles of fusion and fission, referred as mitochondrial dynamics, to maintain their physiological functions (Tilokani et al., 2018). Mitochondrial fusion, whereby one mitochondrion is produced from the union of two mitochondria, promotes the reduction and dilution of damage in each mitochondria by mixing their matrix contents for complementation (Byrne et al., 2019). *MFN1*, *MFN2*, and *OPA1* encode key factors for mediating mitochondrial fusion (Hall et al., 2014). Alternatively, mitochondrial fission, whereby one mitochondrion is divided into two daughter mitochondria, not only regulates the amount and distribution of mitochondria but also plays a role in disposing their deteriorated contents by autophagy or mitophagy. Mitochondrial fission is regulated mainly by *DRP1* expression (Balog et al., 2016; Byrne et al., 2019).

Mitochondrial quality control is necessary in highly structured skeletal muscles (Favaro et al., 2019). In the PMs of broilers, the expression of these mitochondrial dynamics-related genes showed strong positive correlation with that of *VEGFA*, suggesting that active and continuous mitochondrial dynamics affords the maintenance and clearance of mitochondria damaged by hypoxia at the early stage of WB, during which active angiogenesis occurs. The negative correlation between mitochondrial dynamics-related genes with histopathological indices, especially that of *MFN1* and *MFN2* with FA and/or CM, indicated that WB pathology deteriorates in conjunction with reduced mitochondrial dynamics, especially fusion.

The series of mitochondrial fusion, fission, and mitophagy is integrated into the sequential mechanism of damaged mitochondria maintenance; i.e., the reorganization and segregation of damaged mitochondrial contents into daughter mitochondria that are eliminated by autophagy and/or mitophagy (Twig et al., 2008). In particular, the muscle specific loss of *Drp1* in mouse resulted in myofiber death, atrophy, and degeneration of skeletal muscles, involving inhibition of autophagy and mitophagy (Favaro et al., 2019). The strong positive correlation between the expression of mitochondrial dynamics- and autophagy-/mitophagy-related genes observed in the present study suggested that the maintenance of mitochondrial health is highly controlled by this sequential mechanism in broiler skeletal muscles as well. Specifically, whereas the active maintenance of mitochondrial health by their fusion, fission, and autophagy/mitophagy is triggered in response to the hypoxia at the early stage of WB, the acquired resistance to the HIF1 signaling under conditions of chronic hypoxia appears to lead to the attenuated regulation of mitochondrial maintenance by appropriate mitochondrial dynamics and autophagy/mitophagy. In addition, the broilers cannot voluntarily regulate energy balance and exhibit hyperphagia, resulting in excessive accumulation of energy (Richards, 2003). In the situation of decreased energy demand and increased supply, mitochondria progress through fission and fragmentation to adapt to the nutrient excess, which is associated with impaired mitochondrial function (Liesa and Shirihai, 2013). The damaged mitochondria in PMs mediated by excess nutrients may therefore also be screened out through the process of mitochondrial clearance in broilers.

**FIGURE 4:**
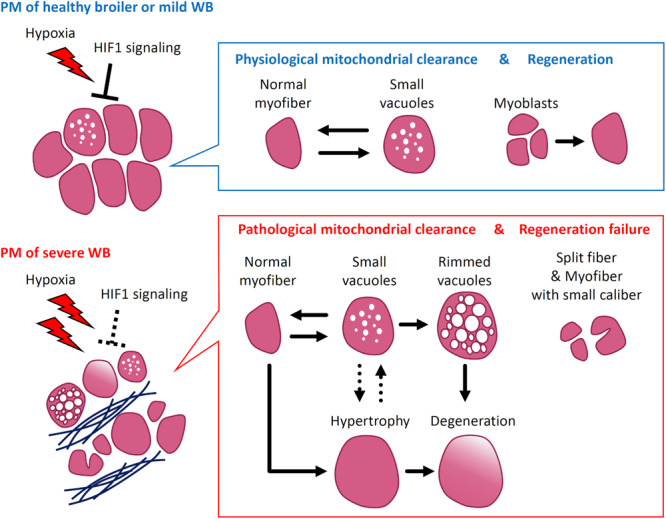
Scheme of wooden breast syndrome (WB) pathogenesis focusing on mitochondrial clearance and myofiber pathology. In pectoralis major muscles (PM) with no or mild WB, the adaptation to hypoxia by HIF1 signaling induces physiological mitochondrial clearance mediated by mitochondrial fusion, fission, and autophagy/mitophagy, which corresponds with the appearance of myofibers with small vacuoles. At this stage, PM repair appears to occur through the differentiation of myoblasts into myofibers. Alternatively, in the muscles exhibiting severe WB, the acquired resistance to hypoxia alters the regulation of mitochondrial clearance. The accumulated damage in mitochondria exacerbates the various pathological characteristics such as myofibers with rimmed vacuoles, along with their hypertrophy and degeneration. Under severe hypoxia, the regeneration of myofibers fails, which corresponds with the frequent appearance of split fibers and myofibers with small caliber. HIF1, hypoxia-inducible factor 1.

In conclusion, our findings demonstrate that the PMs of broilers support the mechanism of physiological mitochondrial clearance, which is defined as the mechanism for maintaining mitochondria damaged by hypoxia as mediated by their continuous fusion, fission, and autophagy/mitophagy. In broilers in which the muscle is constantly exposed to hypoxia from early life owing to a genetic background that facilitates rapidly increasing PM mass, the HIF1-mediated response to hypoxia induces physiological mitochondrial clearance. However, the repeated and chronic hypoxia in the PMs weakens HIF1 signaling, exacerbating the oxygen deficiency and reducing physiological mitochondrial clearance. Eventually, the accumulation of damaged mitochondria would induce myofiber hypertrophy, degeneration, and reduced regeneration in the PMs along with fibrosis, reflecting the pathological characteristics of severe WB (Figure 4). This study revealed the essential role of physiological and pathological mitochondrial clearance in skeletal muscle in the pathogenesis of severe WB. Thus, our findings suggest that suppressing hypoxic stress from the initial stage may be crucial to prevent the development of severe WB.

## Data Availability Statement

The raw data supporting the conclusions of this article will be made available by the authors, without undue reservation, to any qualified researcher.

## Ethics Statement

The animal study was reviewed and approved by the Rakuno Gakuen University Institutional Animal Care and Use Committee.

## Author Contributions

MarH, YH, YW, and MakH acquired the data. MarH, YW, and MakH analyzed the data. MarH, TI, and TW interpreted the results. TK, TT, TI, and TW designed the study. NT and HU provided technical assistance with transmission electron microscopy. MarH and TW wrote the first draft of the manuscript. All authors approved the final version of the manuscript.

## Conflict of Interest

The authors declare that the research was conducted in the absence of any commercial or financial relationships that could be construed as a potential conflict of interest.
