# Supplementary material for: Physiological and Pathological Mitochondrial Clearance Is Related to Pectoralis Major Muscle Pathogenesis in Broilers With Wooden Breast Syndrome
Source: Front Physiol. 2020 Jun 16;11:579. doi: 10.3389/fphys.2020.00579 (PMC7308532; doi:10.3389/fphys.2020.00579)
Supplement: Supplementary file 1 [file Table_1.docx]

Supplementary Material

**Supplementary TABLE 1 |** Clinical data of broilers during the observation period.

| Sample No. | Stage | Body weight (kg) | | | Weight gain ratio  (15 days:50 days) | Wing lift examination | | |
| --- | --- | --- | --- | --- | --- | --- | --- | --- |
|  |  | 15 days | 44 days | 50 days |  | 15 days | 44 days | 50 days |
| 1 | 1 | 0.49 | 3.11 | 3.41 | 6.96 | UA | A | A |
| 2 | 1 | 0.57 | 3.2 | 3.56 | 6.25 | UA | UA | UA |
| 3 | 1 | 0.59 | 3.38 | 4.08 | 6.92 | UA | UA | UA |
| 4 | 1 | 0.61 | 3.16 | 4.34 | 7.11 | UA | A | A |
| 5 | 1 | 0.55 | 3.4 | 4.18 | 7.60 | UA | A | A |
| 6 | 1 | 0.23 | 2.66 | 3.38 | 14.70 | UA | UA | A |
| 7 | 2 | 0.48 | 3.03 | 3.68 | 7.67 | UA | A | A |
| 8 | 2 | 0.56 | 3.29 | 3.78 | 6.75 | UA | A | A |
| 9 | 2 | 0.57 | 3.85 | 4.82 | 8.46 | UA | A | A |
| 10 | 2 | 0.41 | 3.58 | 4.48 | 10.93 | UA | A | A |
| 11 | 2 | 0.6 | 2.74 | 3.56 | 5.93 | UA | UA | A |
| 12 | 3 | 0.48 | ND | 3.48 | 7.25 | UA | ND | UA |
| 13 | 3 | 0.56 | 3.56 | 4.26 | 7.61 | UA | A | A |
| 14 | 3 | 0.48 | 3.34 | 4.08 | 8.50 | UA | UA | UA |
| 15 | 3 | 0.53 | 3.23 | 4.34 | 8.19 | UA | A | A |
| 16 | 3 | 0.42 | 3.02 | 3.82 | 9.10 | UA | A | A |
| 17 | 3 | 0.54 | 3.81 | 3.58 | 6.63 | UA | A | A |
| 18 | 4 | 0.63 | 3.9 | 4.78 | 7.59 | UA | A | A |
| 19 | 4 | 0.52 | 3.55 | 4.28 | 8.23 | UA | A | A |
| 20 | 4 | 0.55 | 4.08 | 4.68 | 8.51 | UA | A | A |
| 21 | 4 | 0.45 | 3.41 | 4.12 | 9.16 | UA | A | A |
| 22 | 4 | 0.5 | 3.18 | 3.91 | 7.82 | UA | UA | UA |
| 23 | 5 | 0.4 | 2.93 | 3.55 | 8.88 | UA | A | A |
| 24 | 5 | 0.55 | 3.34 | 3.98 | 7.24 | UA | A | A |
| 25 | 5 | 0.56 | 2.93 | 3.38 | 6.04 | UA | A | A |
| 26 | 5 | 0.58 | 4.01 | 4.98 | 8.59 | UA | A | A |
| 27 | 5 | 0.54 | 3.37 | 4.23 | 7.83 | UA | UA | A |
| 28 | 5 | 0.48 | 3.05 | 3.73 | 7.77 | UA | A | A |
| 29 | 5 | 0.61 | 4.18 | 5.08 | 8.33 | UA | A | A |
| 30 | 5 | 0.57 | 3.00 | 3.56 | 6.25 | UA | A | A |
| 31 | 5 | 0.47 | 2.97 | 4.69 | 9.98 | UA | A | A |
| 32 | 6 | 0.42 | 3.53 | 4.32 | 10.29 | UA | A | A |
| 33 | 6 | 0.51 | 3.98 | 4.00 | 7.84 | UA | A | A |
| 34 | 6 | 0.48 | 3.02 | 3.78 | 7.88 | UA | UA | A |
| 35 | 6 | 0.66 | 4.08 | 4.78 | 7.24 | UA | A | A |

UA: unaffected birds, A: affected birds, ND: no data.

**Supplementary TABLE 2 |** Spearman’s correlation coefficient (ρ) between muscle histopathological indices and body weight during three points of age or weight gain ratio from 15 to 50 days.

|  |  | Histopathological index | |
| --- | --- | --- | --- |
|  |  | FA | CM |
| Body weight at 15 days (n = 35) | ρ | −0.063 | 0.17 |
|  | *P* | 0.7178 | 0.3370 |
| Body weight at 44 days (n = 34) | ρ | 0.16 | −0.038 |
|  | *P* | 0.3768 | 0.8301 |
| Body weight at 50 days (n = 35) | ρ | 0.25 | 0.11 |
|  | *P* | 0.1551 | 0.5346 |
| Weight gain ratio (15 days:50 days) (n = 34) | ρ | 0.25 | −0.056 |
|  | *P* | 0.1531 | 0.7492 |

FA, fibrotic area; CM, circularity of microfibers

**Supplementary TABLE 3 |** Mean and standard error of the mean (SE) of the fibrotic area (FA) in the muscle and circularity of myofibers (CM).

|  | UA | A | *P* |
| --- | --- | --- | --- |
| FA (%) (n = 35) | 21.7± 0.7 | 30.6 ± 0.7 | 0.0512 |
| CM (n = 35) | 0.67 ± 0.02 | 0.70 ± 0.007 | 0.107 |

Data between unaffected (UA) and affected (A) birds were compared using the Student t test (*P* < 0.05).
